# Validation of a straightforward high performance liquid chromatographic method for morphine quantitation

**DOI:** 10.1186/s41935-017-0003-0

**Published:** 2017-07-18

**Authors:** Kar-Weng Chan

**Affiliations:** Research and Instrumentation Unit, Department of Chemistry Malaysia, Ministry of Science, Technology and Innovation (MOSTI), Petaling Jaya, 46661 Malaysia

**Keywords:** Morphine, Illicit Heroin, HPLC, Quantitative method

## Abstract

**Background:**

Morphine is illegal to use unless it is prescribed by a medical doctor. This compound is commonly abused in the form of illicit heroin.

**Methods:**

An easy-to-use method for determining morphine in illicit heroin using high performance liquid chromatography-photodiode array detector (HPLC-PDA) was developed. With the aid of 50:50 acetonitrile:ammonium formate, this target compound traveling in a pentafluorophenyl (PFP) column was successfully separated from other compounds frequently present in the heroin matrix.

**Results:**

The method was precise with an intra-day relative standard deviation (RSD) ≤ 0.8% and inter-day RSD < 5%. It was able to detect as low as 0.001 mg/mL morphine. The detector’s response was linear (*R* ^2^ > 0.999) and reliable for morphine quantitation from 0.005 up to 1 mg/mL. Through recovery studies, accuracy of the method was averagely estimated to be 89.54–101.91%.

**Conclusions:**

This method saves time in terms of mobile phase preparation and cuts cost by excluding additional purchase of expensive chemicals. More importantly, it proves to be able to determine the target analyte with sufficient accuracy and precision.

## Background

Morphine (C_17_H_19_NO_3_) is medically adopted as a licit pain killer to control severe pain in patients. The danger of this compound is highly attributed to its detrimental effects of habituation and addiction (Weill & Weiss 1951). Since its emergence, morphine has been exploited in the form of illicit heroin (where diacetylmorphine can naturally be hydrolyzed to morphine under improper storage conditions) to create self-pleasure. Due to this reason, opiates are globally controlled through the 1961 Convention (UNODC 2003). In Malaysia, illegal consumption and distribution of opiates or their products are unlawful.

To analyze opiates including morphine, several rapid color tests such as Marquis, Mecke and Frohde tests are recommended (UNODC 1998), with Marquis being the most widely adopted spot test for screening purposes. Without chromatographic separation, color responses alone do not provide much information about the drug’s identity. Therefore separation techniques play an imperative role in segregating opiates. Classically, thin layer chromatography (TLC) can separate opiates on a two-dimensional plate and the type of opiate is identified based on the R*f* value. However, TLC is less sought after because it requires at least ninety minutes to complete the analysis (Steenstra & Logtenberg 1977).

For confirmation, analysis by instrumental techniques is mandatory. A huge number of analytical methods have been developed to quantitate morphine related opiates but nearly all of them were purportedly designed for drug profiling (Ravreby 1987; Moros et al. 2008; Walker et al. 1994; Zhang et al. 2004; Kaa & Bent 1986; Narayanaswani 1985; Chan et al. 2012).

As far as narcotic analysis is concerned, gas chromatography-mass spectrometry (GC) is more superior to other instrumental techniques due to the fact that GC involves little analytical preparation. On a routine basis, a GC column with 5% polarity is desired for universal use (Chan et al. 2012). Unfortunately, morphine is more polar and thus difficult to chromatograph with such a less polar column (Cole & Caddy 1995). Quantitation of this compound by GC can be better performed with derivatization to minimize its polarity (Popa et al. 1998; Neumann 1984). Analysis without derivatization is also possible but the morphine peak was found to be too small (Barnfield et al. 1988), and inconsistent to quantitate (Chan et al. 2012). In addition, adsorptive losses of morphine were also observed (Gough & Baker 1981). This could be due to on-line degradation of the analyte under elevated temperatures at the injector port and transfer line (Klous et al. 2006).

High performance liquid chromatography-photodiode array detector (HPLC-PDA) is an alternative for morphine quantitative work but limitations are inherent. One of the major caveats associated with HPLC is that less routinely used chemicals like hexylamine and octane sulfonic acid sodium may be required to quantitate morphine and other opiates (Lurie & Carr 1986; Lee et al. 2005). Besides, peak fronting and peak tailing of morphine were evident in some studies (White et al. 1983; Baker & Gough 1981). A better peak shape is only obtained, usually accompanied by a tedious preparation of mobile phase (Twitchett 1975).

Prior to this study, evaluation of morphine with a GC method was carried out. Poor precision and insufficient linearity have dismissed this gas phase technique for routine analysis. Subsequently, it was decided to employ HPLC-PDA for this purpose. Although relevant HPLC methods are available elsewhere, this study seeks to simplify an HPLC method that is fit for local sample matrices by minimizing the use of solvents/reagents.

## Methods

### Chemicals and solvents

Morphine hydrochloride and caffeine were respectively purchased from Johnson Matthey Macfarlan Smith (Edinburgh) and Merck (Darmstadt). Codeine and diacetylmorphine were procured from Toronto Research Chemicals (Canada). 6-Monoacetylmorphine was obtained from the Department of Chemistry Malaysia (Malaysia). Ammonium formate, methanol and acetonitrile were supplied by Fisher Scientific (Loughborough). Ultrapure water was generated from the Ultrapure water system at a resistance of 18.2 MΩcm.

### High performance liquid chromatography-photodiode array detector (HPLC-PDA)

Quantitation of morphine was accomplished with a Waters e2695 HPLC Separations Module coupled with a Waters 2996 Photodiode Array Detector. The system was preinstalled with a Kinetex 5u PFP 100A column (150 x 4.6 mm). Separation was achieved by flowing 50:50 acetonitrile:ammonium formate isocratically through the system. Other HPLC conditions are summarized in Table 1.

**Table 1:** Optimized conditions for HPLC

Parameter	Condition
Mobile phase 1 (Bottle C)	Acetonitrile
Mobile phase 2 (Bottle D)	2 mM ammonium formate
Mode of mobile phase	Isocratic 50:50 C:D
Flow rate	1 mL/min
Degasser	Normal
Column temperature	30°C ± 5 °C
Autosampler temperature	20°C ± 5 °C
Injection volume	25 μL
Mode of data acquisition	3D data collection
Scan range	190 to 400 nm
Resolution	1.2
Sampling rate	1.0
UV max detection for quantification	210 nm
Total run time^a^	18–25 min

^a^Maximum run time depends on the nature of the general sample matrix

### Preparation of standard

A desired amount of morphine HCl (e.g. 6 mg for routine calibration; 4 mg for performance checking) was weighed and dissolved with methanol in a 10 mL volumetric flask. The prepared standard was kept in a refrigerator at 5 ± 3 °C for not more than 3 months.

### Preparation of 2mM ammonium formate solution

Approximately 1.89 g of ammonium formate was dissolved with 300 mL water. The solution was filtered and stored in a refrigerator at 5 ± 3 °C for not more than 3 months. From this solution, 20 mL was transferred out and diluted with water to obtain 1 L of freshly prepared salt solution.

### Preparation of sample

Approximately 20–70 mg of a finely ground powder was weighed into a 10 mL volumetric flask to which methanol was added to the mark. The solution was sonicated for 5 min to allow complete dissolution of the solid substance. The solution was then filtered and transferred to a vial, awaiting analysis.

### Partial method validation

The HPLC method’s selectivity was studied using five illicit heroin samples from different sources and a mixture of five compounds (caffeine, morphine, codeine, 6-monoacetylmorphine and diacetylmorphine) frequently present in Malaysian heroin samples (Chan et al. 2012). For precision studies, a sample with a low level of morphine (approximately 0.15 mg/mL) and standard solutions respectively containing an intermediate level (0.3200 mg/mL) and high level (0.5186 mg/mL) of morphine were prepared and analyzed repetitively. These aliquots were decided as such because the target method will adopt 0.51 mg/mL morphine for calibration and 0.32 mg/mL morphine for performance checking. The sample with 0.15 mg/mL morphine on the other hand was aimed at checking signal consistency at low level with the presence of the target sample matrix. Serial dilutions at low levels (0.001, 0.002, 0.004 and 0.008 mg/mL morphine) prepared with solutions containing approximately 12 mg/mL caffeine were experimented to study their ideal peak heights and ultraviolet (UV) spectra in order to determine the limit of detection (LOD) for the method. Its limit of quantification (LOQ) was determined by evaluating the precision of 0.002 and 0.005 mg/mL morphine standards. Nine levels of standard solutions covering 0.005 to 1 mg/mL were prepared in methanol for linearity testing. Three levels of spiking (0.07, 0.22 and 0.44 mg/mL morphine) were prepared and analyzed for 3 days for recovery studies.

## Results and discussion

### System optimization

To achieve separation, different combinations of various solvent systems constituted of methanol, water and acetonitrile coupled with the C18 column in different dimensions were tested but none of them was able to retain morphine in a chromatographically sound manner. A better retention profile was obtained when morphine was allowed to travel with acetonitrile and ammonium formate through a pentafluorophenyl (PFP) column.

For sample dissolution, the principle ‘the like dissolves the like’ must be realized to ensure stable distribution of the analyte in the chosen solvent(s). The polar nature of morphine makes it soluble in any polar solvents such as water, methanol and ethanol. Among all these solvents, water is cheap and has the strongest polarity and thus should be ideally chosen to dissolve the target compound. Unfortunately, this solvent extracted morphine alongside a co-eluting excipient from the illicit heroin matrix. Alternatively, methanol being the second most polar solvent was chosen for this study.

With the presence of an internal standard (IS), area ratio can be employed to obviate certain errors arising from the instrument. A routinely used chemical, 2,2,2 triphenylacetophenone could have been incorporated for this purpose but it was subsequently dismissed because the late eluting opiates tended to interfere with it. Similar to some studies that employed no IS for their quantitative work (Lee et al. 2005; Nogueira et al. 2011), a quantitative method for morphine was developed in the external standard mode. This is justifiable because the method does not involve extraction whereas the signal response (the peak area) is sufficiently high to overcome insignificant errors associated with the overall method. The method validation performance reported thereafter will prove this point.

### Selectivity

**Fig. 1:**
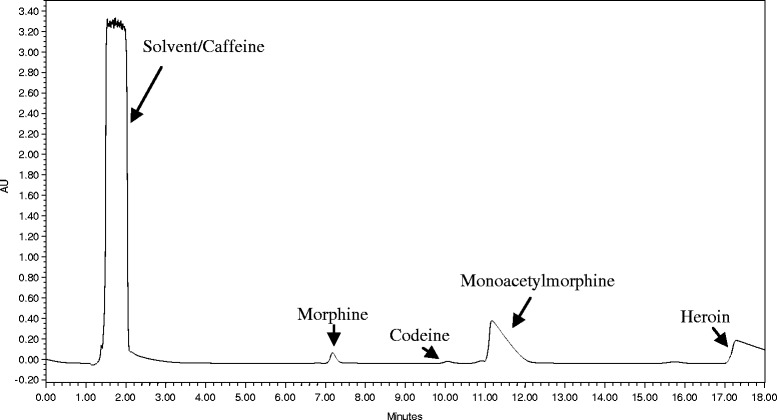
Profile of eluting peaks from an illicit heroin sample

The method’s selectivity was initially tested towards morphine in the presence of four other major compounds (caffeine, codeine, 6-monoacetylmorphine and diacetylmorphine) frequently present in local illicit heroin. The test presented a clear elution order for all the known compounds where morphine eluted much earlier than the other opiates. To rule out interference, selectivity was again checked with five genuine illicit heroin samples which contained even more opiate by-products. There was no noticeable interference observed from the test. Although morphine elutes much earlier, the test with illicit heroin matrices showed that a minimum run time of 18 min (Fig. 1) is mandatory for the all the opiate compounds to purge out to eliminate carry-overs in the subsequent run.

### Precision

Precision is vital to ensure the instrument is able to give off a set of close-by results. Precision of the method was expressed as the percentage relative standard deviation (%RSD) for the morphine peak area collected from repetitive injections. Three prepared aliquots at three different concentration levels (approximately 0.15, 0.3200 and 0.5186 mg/mL morphine) were injected ten times consecutively on the same day to study intra-day precision. The measure was found excellent with an RSD ≤ 0.8%, indicating sufficient consistency of the analyte in the dissolving solvent and mobile phase used in this method.

The study was extended to inter-hour precision by injecting the aliquots once every 4 h, with the last injection made at the 72^nd^ hour. The RSD was found to be less than 2.5%. This substantiates that the samples can be left unattended in the sample chamber for 3 days without incurring significant changes to the target analyte. For inter-day precision, the aliquots were analyzed once over ten different days and they presented an RSD < 5%. The overall precision study infers that peak area alone instead of area ratio is able to provide consistent readings. In terms of precision, the method is fit for the intended purpose (UNODC 2009).

### Limits of detection (LOD) and quantification (LOQ)

**Fig. 2:**
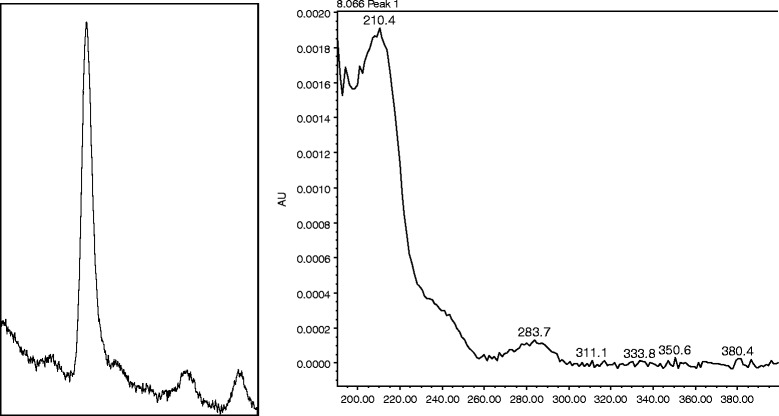
Morphine peak and its UV spectrum at 0.001 mg/mL

In narcotic drug analysis, blank samples are never available. A sample blank can be produced by preparing a cocktail of excipients or using the major diluent. As most local illicit heroin samples are largely constituted by caffeine (about 80%), a caffeine solution (approximately 12 mg/mL) was employed as a blank matrix to prepare four dilutions at low levels (0.001–0.008 mg/mL) to study LOD under the caffeine effect. Each level was analyzed to check for its signal-to-noise (S/N) ratio and the associated UV spectrum. Following this, the LOD was estimated to be 0.001 mg/mL at which level the peak height was found to exceed 3 S/N ratio and the UV spectrum remained undistorted (Fig. 2). In fact, the instrument was able to detect a much lower amount of morphine (below 0.001 mg/mL). Since much of the UV spectrum’s characteristics were lost due to the scarcity of the morphine signal at low levels, the LOD was then decidedly set at the aforementioned value.

As matrix match is impossible for seized materials due to the unavailability of sample blanks, calibration of an instrument is normally performed with standards directly prepared in the target solvent. Owing to this reason, LOQ was determined by analyzing 0.002 and 0.005 mg/mL morphine standards prepared in methanol. These levels were the decision concentration points decided by the laboratory.

The major criterion to determine the LOQ is based on the precision. On this ground, the LOQ was determined to be at 0.005 mg/mL as this level demonstrated a more consistent peak area with an RSD = 6.87% from seven consecutive injections, compared to 0.002 mg/mL which showed a relatively poor precision with an RSD = 14.33%.

### Linearity

**Fig. 3:**
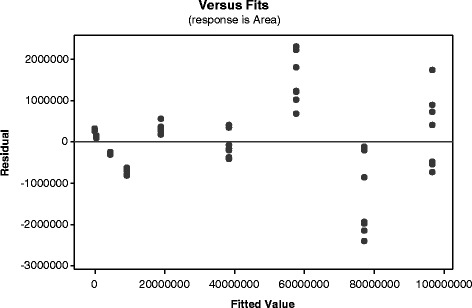
Residual plot with acceptable level of randomness in distribution

Nine levels of morphine standards covering the range from 0.005 to 1 mg/mL were analyzed seven times for linearity testing. The instrument presented a good linear fit with a coefficient of determination, R^2^ = 0.9993 in the peak area versus concentration calibration model. Each level also displayed excellent precision with an RSD < 4% except for the LOQ. The data for this linearity curve were reliable, substantiated by the residual plot in Fig. 3 which illustrates satisfactorily well distributed data points around the zero-line.

For routine calibration, one-point calibration is desirable. Suitability of one-point calibration was assessed by re-plotting the linear curve through the origin (starting from zero to 1 mg/mL) which resulted in an R^2^ = 0.9992. This infers that one-point calibration is sufficient for the intended purpose; choosing any single point along the regression line can approximate multiple point calibration curve for quantitative measurements. To this end, 0.5 mg/mL was tentatively chosen as the target calibration point. In this regard, the previously reported intra-day and inter-day data obtained from the standard containing 0.5189 mg/mL morphine was re-examined for its variance. The independent *T*-test suggested no significant difference in the mean peak areas with a *p*-value = 0.798, whereas Levene’s test displayed equal variances with a *p*-value = 0.072 for both data sets. Therefore the chosen level is sufficiently consistent and thus appropriately adopted as the calibration point.

### Accuracy by recovery

Three different illicit heroin matrices were utilized for recovery testing. Each sample was spiked with 0.07, 0.22 and 0.44 mg/mL morphine. Each resulting aliquot was analyzed in triplicate for 3 days. Percentage recovery was computed following the formula below:

$$ \frac{\mathrm{Measured}\ \mathrm{conc}\ \left(\mathrm{mg}/\mathrm{mL}\right)}{\mathrm{Conc}\ \mathrm{in}\ \mathrm{original}\ \mathrm{sample} + \mathrm{spiked}\ \mathrm{conc}\ \left(\mathrm{mg}/\mathrm{mL}\right)} \times 100 $$

According to Table 2, the method was able to produce a mean recovery from 89.54 to 101.91% from different heroin matrices. Some underestimation was particularly noticeable in Samples A and B at low levels. Such an outcome was probably due to i) the sample matrix rather than the instrument since good recovery was still possible with Sample C, ii) the one-point calibration curve where the calibration point (0.5 mg/mL) was set too high from the measured amount (0.07 mg/mL). For the second issue, more accurate measurement can be achieved by using a larger sample size that renders the measurable amount of morphine close to the calibration point.

**Table 2:** Recovery of morphine from three different sample matrices over 3 days

Spiked level	Sample A	Sample B	Sample C
Low	72.32 ± 4.85%	88.57 ± 3.26%	101.25 ± 0.87%
Medium	94.64 ± 1.68%	95.86 ± 1.43%	99.66 ± 0.92%
High	101.65 ± 2.79%	102.50 ± 1.17%	104.83 ± 0.64%
Mean	89.54 ± 13.58%	95.65 ± 6.32%	101.91 ± 2.40%

## Limitations

No single method is foolproof. HPLC methods do possess analytical threats. It is normal for the signal derived from a high analyte’s amount to present an expanded peak base in HPLC. A large peak will therefore indirectly subsume any impurities eluting in close proximity into its peak, leading to overestimation of the peak area. To eradicate this problem, it is thus recommended to quantify the analyte in low amount (likewise the calibration should be set at a lower point). In the event that large peaks are unavoidable, purity of the morphine peak should not be only assessed by merely checking the UV spectrum. It is also vital to countercheck the match of the peak area ratio obtained by the large morphine peak in question at two different wavelengths (e.g. 210 and 285 nm) against that of the morphine standard.

A noteworthy limitation of this method is that the PFP column requires regular flushing to purge out any contaminants residing within the stationary phase. As time goes by, column clogging does not only result in high backpressure, but also delay elution where the morphine peak tends to take a longer time than certain peaks to elute. In this regard, the method’s selectivity must be verified again.

## Conclusion

Methods designed for simultaneous determination of morphine together with other opiates in illicit heroin are vastly available but they are mostly useful for heroin profiling. To accurately measure morphine alone for prosecution purposes, a designated method is required. In this regard, GC is relatively off putting because it has a much lower sensitivity towards underivatized morphine. HPLC on the other hand involves costly chemicals and tedious mobile phase preparation although it can offer a much lower LOD and better consistency. The present study has developed a much simpler HPLC method without incurring expensive chemicals and laborious mobile phase preparation. The method is straightforward and its performance is sufficiently fit for routine analysis.
